# Electrochemical Characterization of Redox Probes Confined in 3D Conducting Polymer Networks

**DOI:** 10.1002/chem.202103257

**Published:** 2021-11-25

**Authors:** Jochen E. Kuhlmann, Sherri S. Y. Liu, Klaus Dirnberger, Michael Zharnikov, Sabine Ludwigs

**Affiliations:** ^1^ IPOC – Functional Polymers University of Stuttgart Pfaffenwaldring 55 70569 Stuttgart Germany; ^2^ Applied Physical Chemistry Heidelberg University Im Neuenheimer Feld 253 69120 Heidelberg Germany

**Keywords:** click chemistry, conducting polymers, copolymers, electrochemistry, redox species

## Abstract

In this manuscript we present a versatile platform for introducing functional redox species into tailor‐made 3D redox polymer networks. Electrochemical characterization based on cyclic voltammetry is applied to verify the immobilization of the redox species within the conducting networks. Ultimately this strategy shall be extended to (photo)electrocatalytic applications which will profit from the conducting polymer matrix. Soluble precursor copolymers are synthesized via radical copolymerization of vinyltriphenylamine (VTPA) with chloromethylstyrene (CMS) in different ratios, whereas CMS is subsequently converted into azidomethylstyrene (AMS) to yield poly(VTPA‐*co*‐AMS) copolymers. Spin‐coating of poly(VTPA‐*co*‐AMS) on gold electrodes yields thin films which are converted into stable polymer network structures by electrochemical crosslinking of the polymer chains via their pendant triphenylamine groups to yield N,N,N′,N′‐tetraphenylbenzidine (TPB) crosslinking points. Finally, the resulting redox‐active, TPB‐crosslinked films are functionalized with ethynylferrocene (EFc) as a representative redox probe using a click reaction. Main experimental tools are polarization modulation infrared reflection absorption spectroscopy and scan rate dependent cyclic voltammetry. Especially the latter proves the successful conversion and the immobilization of redox probes in the polymer matrix. The results are compared with the reference system of azide‐terminated self‐assembled monolayers on gold substrates, allowing to distinguish between free and immobilized EFc species.

## Graphical abstract

**Tailor‐made 3D conducting polymer** networks containing tetraphenylbenzidine redox units are presented as novel platform for the introduction of tunable amounts of additional functional redox species via polymer analogous click chemistry in films. An in‐depth electrochemical characterization based on cyclic voltammetry shows successful covalent immobilization of the redox species within the conducting networks. Ultimately this strategy shall be extended to (photo)electrocatalytic applications which will profit from the conducting polymer matrix as solid support.

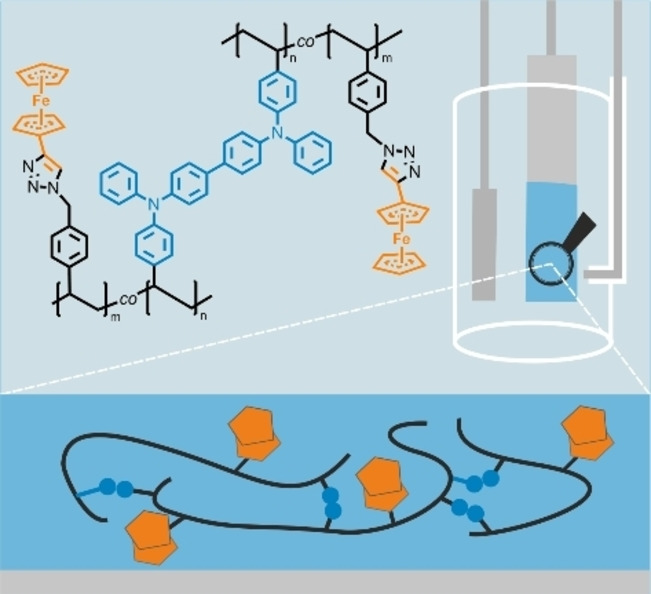

## Introduction

Conducting polymers have attracted much interest due to their versatile solution processability, synthetic variability and lightweight nature which make them attractive materials for a variety of electronic and electrochemical applications. Generally, conducting polymers can be categorized into conjugated and redox systems. Conjugated polymers are typically semicrystalline and allow for electronic conduction by delocalization of charges along the backbone.^[1]^ Redox polymers contain redox‐active groups alongside a non‐conjugated backbone and conduction occurs mainly via hopping of highly localized charges.[^2^, ^3^] A main advantage of redox polymers is that they offer stable redox‐switching and high levels of achievable doping levels which make them particularly interesting for battery and electrocatalytic applications,[^3^, ^4^, ^5^] with examples such as metal complexes (ferrocene, etc.),^[6]^ quinone derivatives,^[7]^ stable radicals like 2,2,6,6‐tetramethylpiperidinyloxyl (TEMPO)^[8]^ and aromatic systems like carbazole.[^9^, ^10^] Recent chemical approaches include also attaching redox units to conjugated polymer backbones yielding conjugated redox polymers.^[11]^

A key feature of conducting polymers is the possibility to create homogenous films via solution processing. Particularly interesting is that *as‐deposited* films can be further chemically modified by polymer‐analogous reactions. For example, they can be stabilized by chemical or electrochemical crosslinking, preserving their as‐deposited morphology which is advantageous for many electronic applications.[^12^, ^13^, ^14^, ^15^] Electrochemical crosslinking is very versatile when conducting substrates are used, resulting even in multifunctional polymer architectures in the case of multifunctional monomers.[^16^, ^17^, ^18^, ^19^]

A relatively simple and reliable method to introduce new functional species in a given polymer film is click chemistry, which is a term describing a set of criteria for chemically versatile and efficient reactions, defined by Sharpless et al. in 2001.^[20]^ A typical *click* reaction is the copper catalyzed 1,3‐dipolar cycloaddition of azides and alkynes (CuAAC) to regioselectively give 1,4‐triazole products.[^20^, ^21^, ^22^] It has been in particular used to decorate self‐assembled monolayers (SAMs) assembled on planar surfaces with various redox probes, such as ferrocene[^23^, ^24^] and porphyrins.[^25^, ^26^] In this context, Chidsey *et al*. used cyclic voltammetry to show successful ferrocene attachment to azide‐terminated SAMs which can be useful for applications such as sensors and electrocatalysis.^[27]^ Barile *et al*. further demonstrated that the modification of an azide‐terminated electrode with alkyne‐terminated metal porphyrins through click chemistry can be applied to the electrocatalytic reduction of CO_2_.[^23^, ^25^, ^27^, ^28^, ^29^]

This strategy is also applicable to polymers. For introduction of redox probes within polymer matrices the respective azide functionality can be brought into the monomer units. This has been done for different kinds of non‐functional polymers and works particularly good for azidomethylstyrene systems. One example from literature reports for example the introduction of various iridium complexes into azidomethylstyrene/*N*‐vinylcarbazole and azidomethylstyrene/styrene copolymers.^[30]^ Others report the usage of CuAAC to create graft polymers,^[31]^ or electropolymerized pyrrole and thiophene^[32]^ onto azidomethylstyrene/styrene copolymer backbones. Particularly interesting is the post‐modification of conducting polymers, such as poly(ethylenedioxythiophene) (PEDOT) which is used for transparent flexible electrodes and electrochemical devices.^[33]^ Conversions of thin films of azide‐bearing PEDOT (PEDOT‐N_3_) were done by Larsen et al. using a CuSO_4_/sodium ascorbate catalytic system.^[34]^ The authors pointed out the benefits of using dimethyl sulfoxide (DMSO) as reaction solvent or as a cosolvent since it promotes swelling of the PEDOT films and dissolving Cu(I) ions which plays an important role in the catalytic cycle.[^35^, ^36^] DMSO has also been reported to accelerate many other CuAAC reactions, even enabling catalyst‐free conversions.^[37]^ In a different catalytic system, films of PEDOT‐N_3_ polymers and copolymers from EDOT‐N_3_ and EDOT were also converted with various organic alkynes by Bäuerle et al. using acetonitrile as the reaction solvent. With (MeCN)_4_Cu(I)PF_6_ as a preformed catalyst which stabilizes ions in polar solvents and elemental copper protecting the catalytic system from external oxidation, redox‐active and electron‐donating ferrocene units could be successfully attached to PEDOT films.[^38^, ^39^] The Ludwigs group reported on branched electropolymerized and chemically polymerized EDOT‐N_3_/2,2’:3’,2’’‐terthiophene (3T) copolymer films to introduce ion functionality.^[40]^ The branched amorphous architectures are inherently suitable for fast counterion exchange reactions upon electrochemical doping.^[41]^

In the case of solution‐processable conjugated polymers Venkataraman et al. proposed to use copolymers of 3‐alkylthiophenes and side‐chain functionalized (4‐(thiophen‐3‐yl)‐but‐1‐inyl)‐triisopropylsilane (3TBT) as linear copolymer systems.^[42]^ Our group used this concept to add additional redox functionality by introducing triphenylamine (TPA) groups to polythiophene backbones.^[43]^ Electrochemical and chemical doping of these copolymers led to concurrent crosslinking of the TPA groups to form N,N,N′,N′‐tetraphenylbenzidine (TPB) units which gave stable films with high electronic conductivities.^[43]^ The systems can be regarded as conjugated redox polymer films.

Homopolymers based on vinyl(triphenylamine) (VTPA) are interesting systems on their own. As mentioned, chemical or electrochemical crosslinking leads to inter‐ and intrachain connections between polymer chains through the formation of TPB units.[^10^, ^44^, ^45^] These crosslinked films are redox‐active and the degree of doping can be fine‐tuned, both chemically and electrochemically. Conductivities as high as 10^−3^ Scm^−1^ could be demonstrated which clearly shows that such crosslinked redox polymer networks can act as conducting layers for transparent electrodes and hole‐transport layers,^[46]^ but may also open a new way to fabricate conducting channels for (photo)electrocatalytic applications. Similar microporous films have been suggested for chemo‐ and biosensing due to their conjugated skeletons and micropores.^[47]^

**Scheme 1:**
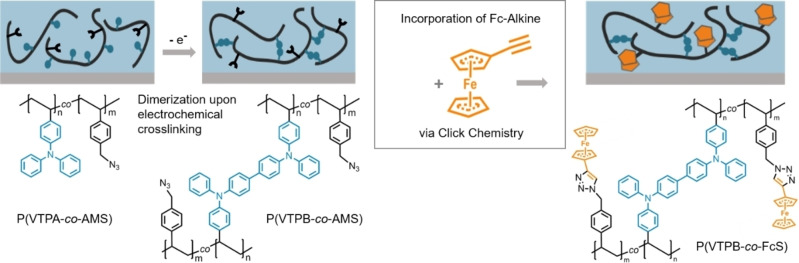
Schematic outline for Fc‐alkyne incorporation via click chemistry into a crosslinked redox polymer film. A solution‐deposited film of P(VTPA‐co‐AMS) is first crosslinked to P(VTPB‐co‐AMS) and then converted into P(VTPB‐co‐FcS).

In the present manuscript we show a straightforward approach to combine the crosslinking strategy of the VTPA polymers with the introduction of additional redox moieties by click chemistry in the VTPA‐based films. In this context, successful copolymerization of VTPA with chloromethylstyrene (CMS) is demonstrated by radical polymerization to yield statistical copolymers (P(VTPA‐*co*‐CMS)) with tunable composition. The CMS moieties in these copolymers can be easily modified, resulting in azide‐bearing copolymers (PVTPA‐*co*‐AMS), which can be subsequently functionalized with suitable alkyne‐bearing molecules via CuAAC as illustrated in Scheme 1.

The strategy is as follows: In a first step, films of the copolymers are deposited by solution‐casting onto electrodes and then electrochemical crosslinking of the films is performed yielding redox‐active polymer films with tetraphenylbenzidine units (PVTPB‐*co*‐AMS). In a second step, ferrocene (Fc) is introduced by click chemistry into the azide monomers to yield a certain amount of Fc within the copolymer films, (PVTPB‐*co*‐FcS). As main characterization method cyclic voltammetry (CV) is applied. The modification with ethynylferrocene (EFc), leading to a Fc‐functionalized conjugated redox polymer, is chosen as a representative test for the approach. Note that EFc is a reversible one‐electron redox system which makes it ideal for a qualitative and quantitative electrochemical analysis through cyclic voltammetry (CV). An additional option is provided by the comparison of the Fc‐functionalized polymer films with the Fc‐substituted self‐assembled monolayers. Ultimately the approach presented in the given study can be extended to electrocatalytically active molecules, with the TPB copolymer films allowing for charge transport and high film stability and the introduced moieties enabling catalytic functionality.

## Results and Discussion

**Figure 1:**
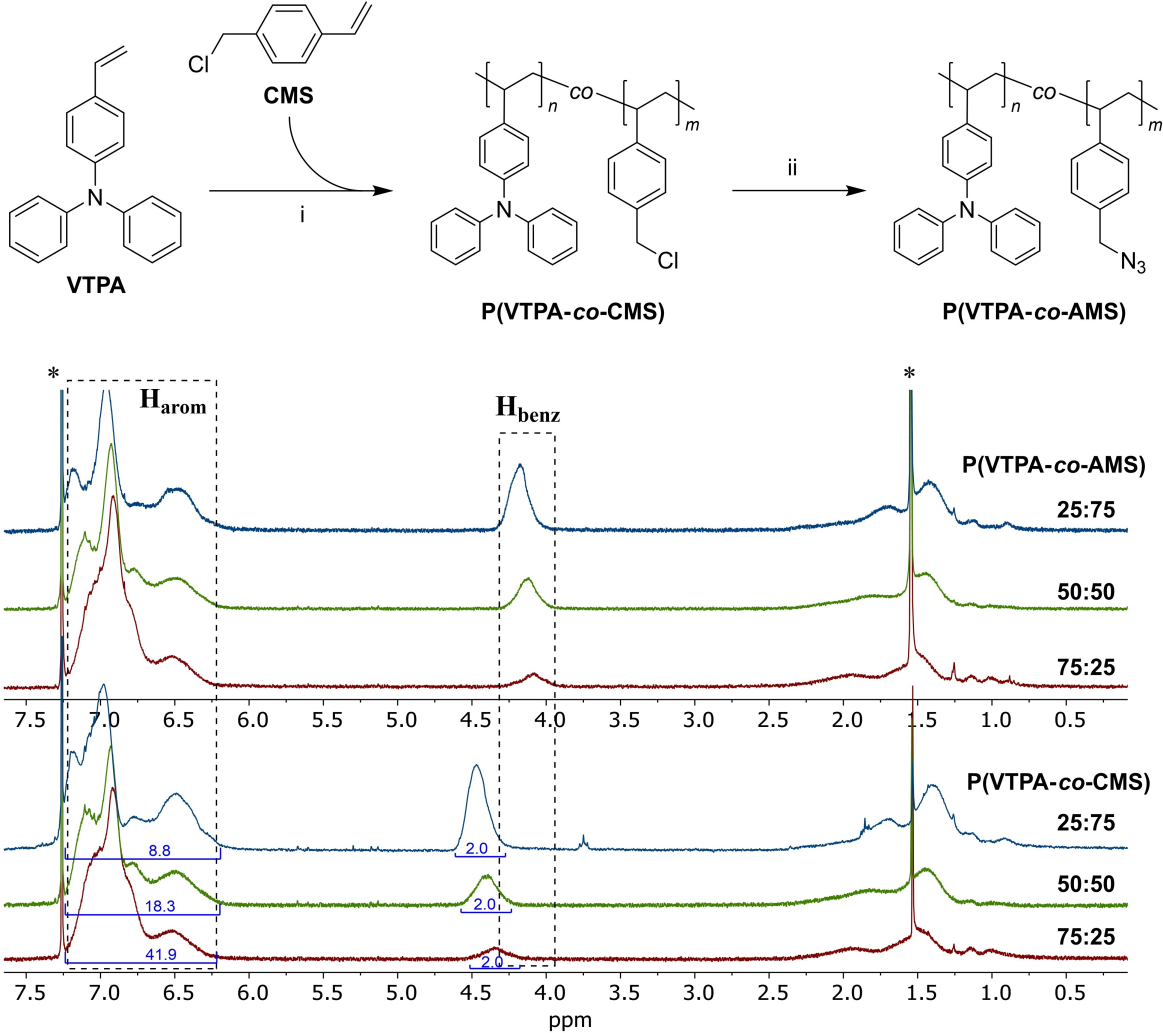
Top panel: Synthetic pathway to P(VTPA‐co‐AMS) copolymers with (i) AIBN, toluene, 70 °C, 48  and (ii) NaN_3_, DMF, rt, 20 h. ^1^H NMR spectra of P(VTPA‐co‐AMS) copolymers (middle panel) and the respective precursors P(VTPA‐co‐CMS) (bottom panel) in CDCl_3_ for several different compositions.

Redox copolymers with varying ratios of triphenylamine (TPA) and azidomethylstyrene (AMS), were copolymerized in different ratios by free radical polymerizations in toluene initiated by azobisisobutyronitrile (AIBN), proceeding with isolated yields of 44–77 %. The reaction conditions were generally based on literature reports,[^48^, ^49^] but were modified to work reliably with different co‐monomer ratios. The individual polymers are named according to the respective feed ratios. These ratios are given in Figure 1 together with corresponding ^1^H NMR spectra from which the actual ratios of repeating units in the polymers were determined. For this determination, signals of the benzylic (H_benz_) and aromatic (H_arom_) protons were used, with the results closely matching the feed ratios. P(VTPA‐*co*‐CMS) copolymers were then further converted with sodium azide by polymer analogous halide‐azide exchange reactions to obtain the corresponding P(VTPA‐*co*‐AMS) copolymers with yields of 76–89 % after thorough workup. This procedure resulted in a clear shift of the benzylic proton signals in the NMR spectra for all three representative compositions in Figure 1, with no residual peaks at the original positions, suggesting a complete conversion. For molecular weight and polydispersity characterization size exclusion chromatography was performed, Figure S1. Mainly 4 polymer batches are characterized: P(VTPA‐*co*‐AMS) 75 : 25 with $\bar{M_{n}}$ =5200 gmol^−1^ and *Ð*=2.34, 50 : 50 with $\bar{M_{n}}$ =12600 gmol^−1^ and *Ð*=2.52 and two batches at 25 : 75 with $\bar{M_{n}}$ =9500 gmol^−1^/*Ð*=2.75 and $\bar{M_{n}}$ =6000 gmol^−1^/*Ð*=1.92. Infrared spectroscopy of all 3 copolymer ratios can be found in Figure S2.

**Figure 2:**
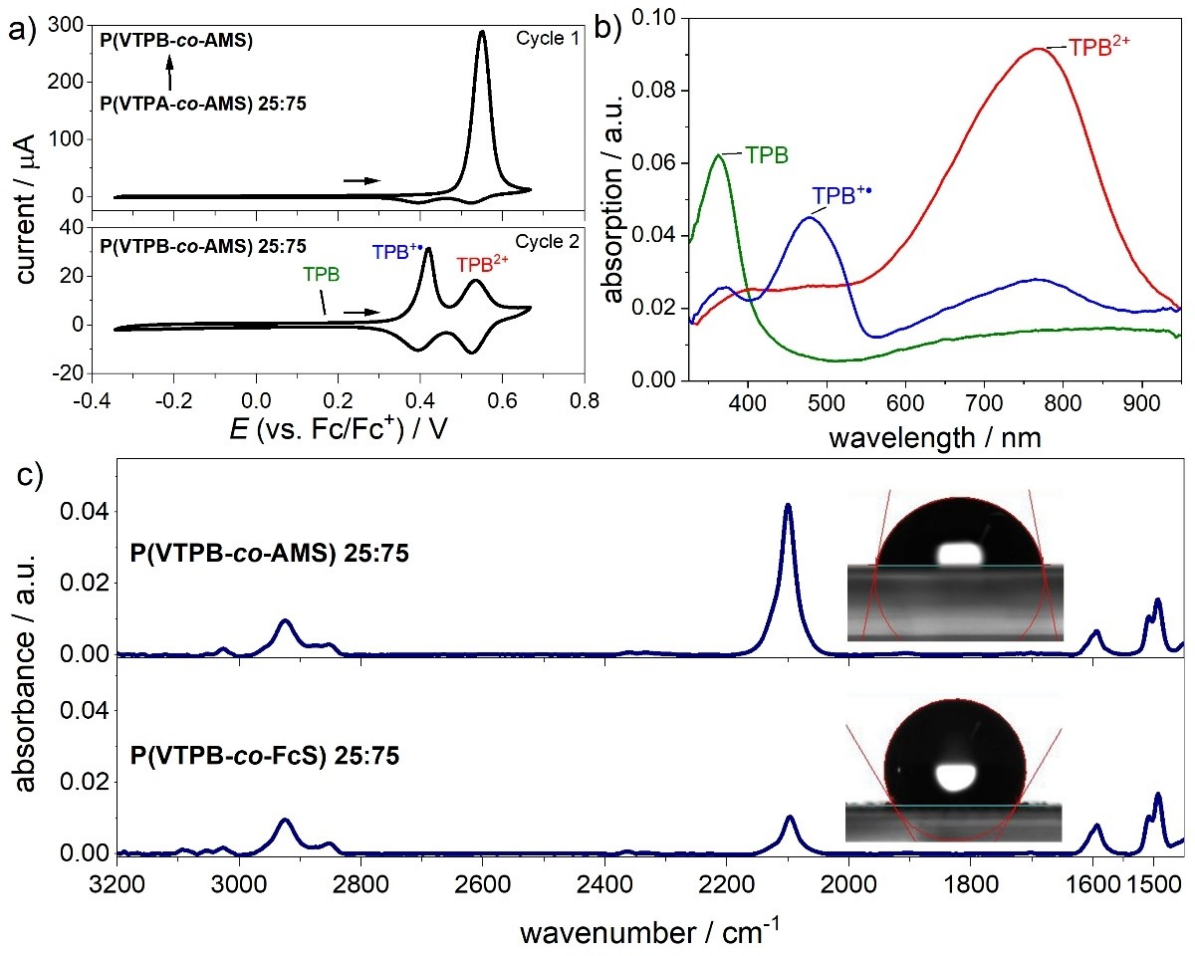
a) First (top) and second (bottom) cycle of the electrochemical crosslinking of a 25 : 75 copolymer film on a gold coated substrate. b) UV‐vis absorption spectra taken during the course of an in situ spectroelectrochemistry experiment of a film of a 25 : 75 polymer batch on ITO, during the 3^rd^ forward cycle after crosslinking, corresponding to the neutral, radical cation and dication TPB redox species. c) PM‐IRRAS of crosslinked polymer films before (top) and after (bottom) introduction of ethynylferrocene. Characteristic azide bands are highlighted. Inserts show associated increase in water contact angle.

Films of the copolymers were prepared via spin‐coating from 5 gL^−1^ toluene solutions on gold and indium tin oxide (ITO) coated glass electrodes. The polymer films were then electrochemically crosslinked via cyclic voltammetry (CV) (Figure S3). Representative CVs are given in Figure 2a); they were carried out under argon atmosphere with gold electrodes at scan rates of 50 mVs^−1^ in acetonitrile/0.1 M tetrabutylammonium hexafluorophosphate (TBAPF_6_). All potentials are referenced against the ferrocene/ferrocenium redox couple, *E*_*1/2*_ vs. Fc/Fc^+^. A schematic explanation of the crosslinking process is presented in Scheme 1. In the first cycle CV, a large anodic wave can be seen with a peak potential at 0.55 V. This wave is indicative of charged radical TPA cations which are unstable due to high spin density at the *para* position,^[10]^ ultimately leading to the irreversible formation of dimerized N,N,N′,N′‐tetraphenylbenzidine (TPB) units.[^44^, ^45^] These TPB units represent redox‐active sites themselves and can be reversibly oxidized, as can be seen in the second cycle. Oxidation of the TPB units proceeds via radical cations TPB^.+^ first, with a half‐wave potential of *E* ^1/2^ ∼0.41 V. Further oxidation leads to dications TPB^2+^ with a half‐wave potential of *E* ^1/2^ ∼0.53 V. The half wave potentials agree well with the data for the corresponding PVTPA homopolymers.^[46]^ Furthermore, we demonstrated that the polymers are conducting in the charged states, with a maximum conductance when both, radical cations and dications, are prevalent following a mixed valence conductivity model.

Figure 2b presents characteristic UV‐visible absorption spectra recorded during in situ spectroelectrochemical measurements where absorption is measured as function of potential in a CV during the 3^rd^ forward cycle (more details in Figure S4). These measurements were performed on films prepared from chloroform solutions on ITO coated glass slides. Distinct absorption spectra of the polymer for the three oxidation states of TPB can be identified with maxima at 362 nm for neutral TPB, at 476 nm for TPB^.+^, and at ca. 771 nm for the fully oxidized TPB^2+^. These values are in agreement with the literature values for the homopolymers.^[46]^

Crosslinked films on gold substrates were subsequently functionalized with EFc using *click* chemistry. For this purpose, the substrates were submerged for 3 days in a DMSO solution containing the reagents under nitrogen atmosphere. Figure 2c) exemplarily shows the polarization modulation infrared reflection absorption spectroscopy (PM‐IRRAS) spectra of the 25 : 75 polymer film before (top) and after (bottom) the introduction of EFc (the spectra of the other copolymers can be found in Figure S5). Characteristic is the strong band at 2100 cm^−1^ representing the azide groups in the copolymer film. A strong reduction in the intensity of this band can be observed after the reaction, which indicates a high conversion in the films. This band does not however disappear completely which means that not all azide moieties have reacted with EFc. This behavior, also observed by others,^[34]^ can be tentatively explained by the limited accessibility of the azide sites. A further evidence for the introduction of EFc in the polymer films is provided by water contact angle data (see the inserts in Figure 2c) increasing from (78.5±1.2)° to (130.1±5.9)°, in agreement with the introduction of non‐polar Fc groups.

The final evidence is provided by the CV data for the polymer films which were compared with the reference data for free Fc and Fc‐substituted SAMs assembled on planar gold substrates. The latter SAMs were fabricated by clicking EFc to a 11‐azidoundecanethiol self‐assembled monolayer prepared by a standard protocol from literature.^[24]^ The click reaction occurred with nearly 100 % efficiency, as evidenced by the PM‐IRRAS data, Figure S6.

For reference experiments a three‐electrode set‐up was used consisting of a coiled platinum wire (*ø*=0.5 mm) counter electrode (CE), a (true) Ag/AgCl in 3 M KCl reference electrode (Metrohm), and either the azide‐ or Fc‐terminated SAM on Au as the working electrode. The aqueous electrolyte was a 1 M solution of perchloric acid (HClO_4_, 70 %, Sigma Aldrich) dissolved in a solvent mixture of 60 % volume of Millipore water (18.2 MΩ) with 40 % volume of methanol (p.a).

**Figure 3:**
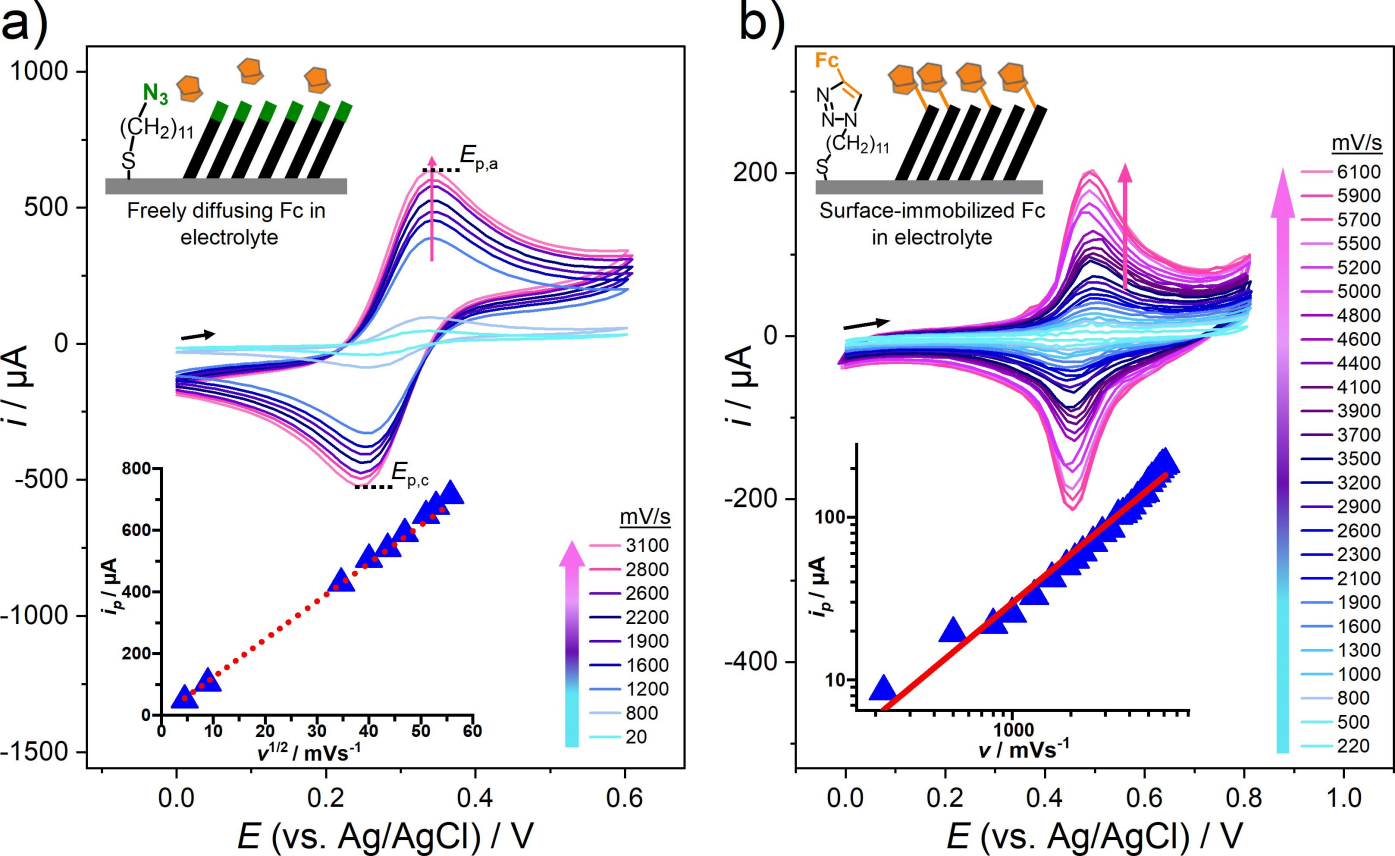
CVs taken in aqueous electrolytes at different scan rates for a Fc‐freely diffusing system (a) and an Fc‐ immobilized system, mimicked by the Fc‐terminated SAM on gold (b). Inset of a) shows the linear relation between i_p_ and ν^1/2^ for the freely diffusing case. Inset of b) shows a linear relation between i_p_ and v for the surface‐immobilized Fc electrode case.

The first reference case corresponded to a freely diffusing system, mimicked by the redox probe molecule ferrocenemethanol (FcMeOH) dissolved in the aqueous electrolyte and the working electrode represented by the azido‐SAM modified gold. The respective CVs in Figure 3a give an *E* ^1/2^ ∼0.3 V vs. Ag/AgCl and show increasing peak current for the anodic (*E_pa_*) and cathodic (*E_pa_*) redox waves with increasing scan rates. A plot of the peak current (*i_p_*) against the square‐root of the scan rate (*v* ^1/2^) is presented in the inset of Figure 3a. It shows a linear relationship between *i_p_* and *v* ^1/2^ which closely follows the Randles‐Sevcik equation for a diffusion‐limited system.^[50]^

The second reference case corresponded to Fc moieties immobilized on the surface of the working electrode as the terminal groups of the SAM, and this system gave an *E* ^1/2^ of 0.5 V vs. Ag/AgCl. The CVs of this Fc‐immobilized system acquired at different scan rates are shown in Figure 3b. The corresponding relationship between the peak currents and scan rate of a Fc‐immobilized system is given in the inset of Figure 3b. An increasing scan rate from 220 mV/s to 6100 mV/s resulted in an increase of the anodic peak current up to ∼200 μA and the dataset follows closely the linear relationship of *i_p_* and *v* which is described by a Langmuir adsorption isotherm.[^24^, ^51^, ^52^] This behavior is distinctly different from that of the free Fc which suggests that the relationship between the peak currents and scan rates can be used to differentiate between the two cases of immobilized and freely‐diffusing Fc‐species.

A further parameter which allows to distinguish the Fc‐freely diffusing case (Figure 3a) from the Fc‐immobilized case (Figure 3b) is the specific shape of the CV. In the Fc‐freely diffusing system, the peak separation between the anodic (*E*_p,a_) and cathodic (*E*_p,c_) peak varies from 90 mV at 50 mV/s to 100 mV for 3100 mV/s. In contrast, the Fc‐immobilized system shows an invariable peak separation of only 30 mV for all scan rates, which is within the theoretical value of 59/*n* mV (at 25 °C) for a totally reversible redox system.^[51]^

This behavior can be attributed to Fc being fully oxidized to Fc^+^ and reduced back to Fc with an almost negligible mass‐transfer limitation in the immobilized case. Thus, the forward scan mirrors the backward scan of the adsorbed electrochemical system, and for an ideal Nernstian reversible system it follows the Langmuir isotherm, $E_{pa}=E_{pc}$ .[^24^, ^50^, ^51^, ^53^] Furthermore, for the Fc‐immobilized system, the peak envelope is overall narrower compared to the freely diffusing case. Thus, one is able to use both the shape and dependence of the peak current on the scan rate for differentiating between immobilized and freely diffusing redox species.

**Figure 4:**
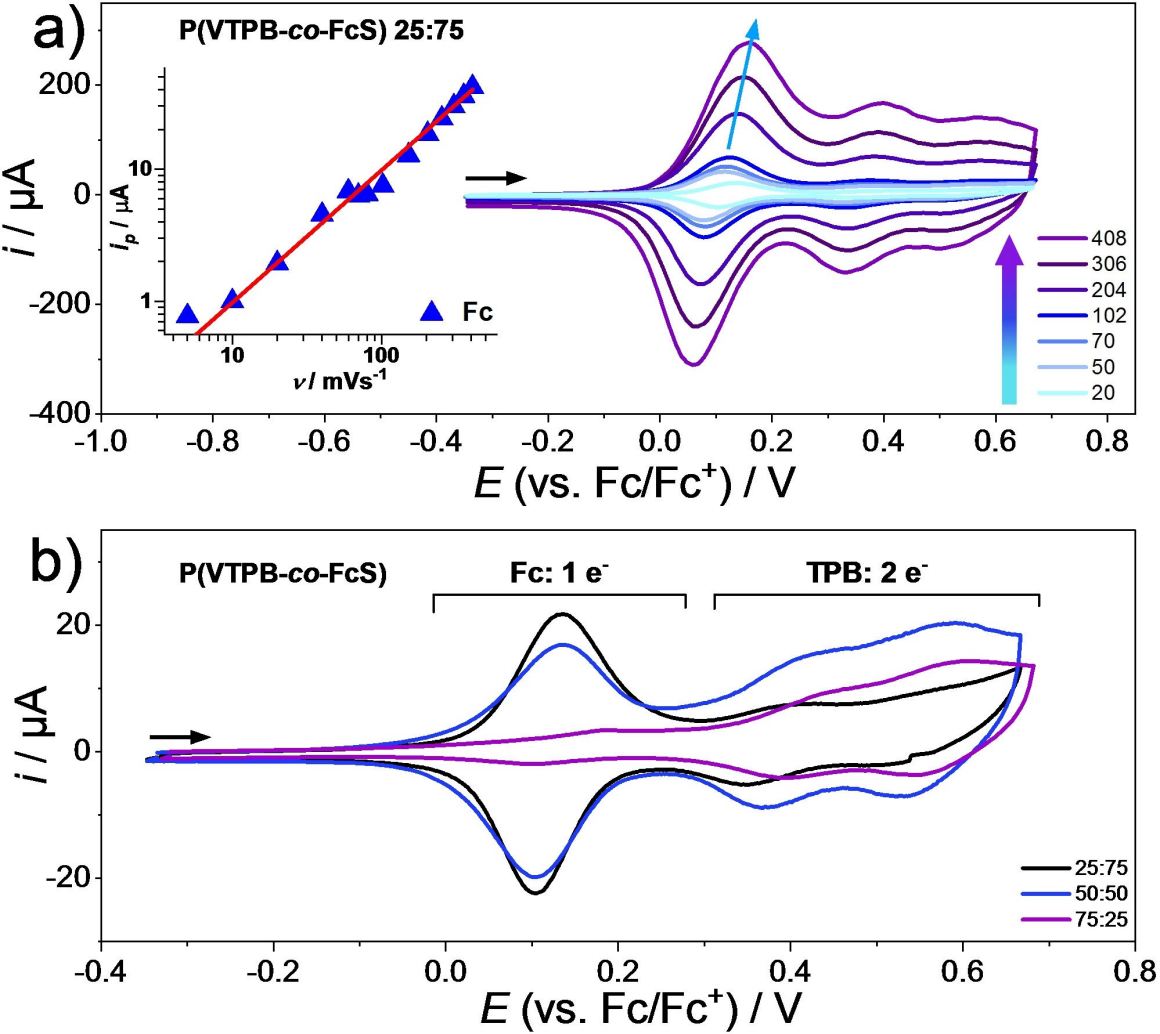
a) CVs taken at different scan rates for the 25 : 75 PVTPB‐co‐FcS copolymer film on a gold electrode, measured in TBAPF_6_ / acetonitrile electrolyte. Inset: Corresponding scan rate dependencies for the Fc redox peak current i_p_ for the forward scans. There is a linear relationship of i_p_ vs. v. b) CVs taken at 20 mVs^−1^ for the copolymer films with 25 : 75, 50 : 50 and 75 : 25 ratios.

These criteria were then applied to the characterization of the crosslinked P(VTPB‐*co*‐FcS) films containing the immobilized ferrocene species. Measurements were done in a standard three electrode set‐up as described above but with a Ag/AgCl pseudo‐reference electrode. The electrolyte solution consisted of 0.1 M tetrabutylammonium hexafluoro phosphate (TBAPF_6_) in acetonitrile. All measurements were referenced against the Fc/Fc^+^ redox pair. The respective first cycles measured at different scan rates for the copolymer system with 25 : 75 copolymer ratio are displayed in Figure 4a. For the full datasets, including the 50 : 50 and 75 : 25 systems, we refer to Figures S7 and S8. Looking at Figure 4a, the first apparent observation is that in addition to the TPB waves at around $E_{1/2}=0.31\;\;V$ and $E_{1/2}=0.49\;\;V$ (referred to a scan rate of 50 mVs^−1^), there is a new wave around $E_{1/2}=0.11\;\;V$ , manifesting the general presence of Fc. For the ferrocene half‐wave peak separation, an increase from 28 mV at 20 mVs^−1^ to 100 mV at 408 mVs^−1^ is observed. From comparing the signal intensities of Fc and the TPB oxidation waves the different shares of ferrocene in the respective copolymers become visible. We note that the shape of the CVs of freely‐diffusing Fc when using P(TPB‐*co*‐AMS) as working electrode look significantly different from the immobilized case (Figure S9), but resemble the ones for the freely‐diffusing case on the azode‐SAMs (Figure 3a).

For evaluation of scan rate dependence for the immobilized P(VTPB‐*co*‐FcS) films, cycles were run with scan rates between 20 mVs^−1^ and 408 mVs^−1^; the respective data are presented in Figure 4a and Figure S7. Fc waves were characterized by picking the peak values of every first forward cycle from the CVs. Logarithmic *i*‐*ν* plots of all copolymers closely follow a linear relationship with a slope of about 1. An analogous depiction of linear scan rate dependency of peak current for the TPB^2+^ redox waves can be found in the Supporting Information (Figure S10). These data clearly show that the ferrocene is part of the redox polymer network system, generating a multi‐redox conjugated polymer.

To allow a quantitative evaluation of the multi‐redox behavior, the CVs of the different P(VTPB‐*co*‐FcS) copolymer films taken at a low scan rate of 20 mV/s were considered (Figure 4b). The analysis is based on the assumption that at low scan rates the integrals of the current in the forward scan of the CV can be used to evaluate the charge relation for the Fc (1 e^−^ oxidation) and TPB (2 e^−^ oxidation) redox units in the copolymer films, see Figure S11. From the molar ratios of the monomer units in P(VTPA‐*co*‐AMS), the theoretical charge ratios for the oxidation of the TPB and Fc entities were calculated as 0.67, 2 and 6 for the 25 : 75, 50 : 50 and 75 : 25 copolymers, respectively. From the integration over the two oxidation waves in the CVs the experimental charge ratios were estimated as ∼1.0 (25 : 75), ∼2.1 (50 : 50) and ∼6.4 (75 : 25). Especially for the 50 : 50 and 72 : 25 copolymer films, the theoretical values fit very well to the experimental ones. This means that the click chemistry to incorporate ferrocene into the copolymer films worked very efficiently, with ferrocene reacting with nearly all available azide groups.

Only for the 25 : 75 copolymer films, the theoretical and experimental values for the charge ratio do not fit perfectly, which suggests incomplete conversion of the click reaction. One might attribute this incompleteness to the fact that the films can act in a partially insulating manner, especially in the case of higher AMS contents, which is the situation in the 25 : 75 copolymer film.

It should be noted that the calculations and the observation that the shape and potential values of the Fc half‐waves deviate to a degree between the different copolymers might be attributed to overlapping redox waves on the one hand and electronic interactions between the Fc and the TPB units on the other hand. However, the peak current versus scan rate plots follow the expected linear relationship in all cases and redox activity remained stable during the experiments, reminiscent of scan rate dependencies of other redox‐active polymers in literature.^[54]^

## Conclusions

In this work, we demonstrate a straightforward pathway to the fabrication of a highly versatile P(VTPA‐*co*‐AMS) copolymer system which can be tailored with regards to the number of functional (AMS) groups by adjusting the co‐monomer ratio. Monomer feed ratios of 25 : 75, 50 : 50 and 75 : 25 were reliably employed. By electrochemical crosslinking in thin films of these polymers, we produced network structures stabilizing these films, thus allowing their subsequent modification by polymer analogous CuAAC click chemistry with a wide range of reactants. As a representative test reactant, redox‐active ethynylferrocene was coupled to both the copolymer films and to planar azide‐terminated SAMs serving as a reference system. Successful conversion was first evidenced by PM‐IRRAS and water contact angle goniometry and further assessed electrochemically, together with redox activity and stability, using scan rate dependent cyclic voltammetry. The reference data allowed us to understand differences in CVs between diffusing and covalently bound redox molecules, relying on the voltammogram shape and the relationship between the peak current *i_p_* and scan rate *ν*.

Relative intensity of ferrocene redox activity compared to that of the polymer TPB redox processes was in accordance with the different shares of EFc in the polymers. Peak current to scan rate relationships showed a linear dependence as is expected for redox active polymers in case of successful covalent binding.

These results pave the way to the introduction of catalytic functionalities and ionic groups into 3D polymer structures and polymer thin films.

## Experimental Section

All chemical were used as received, unless stated otherwise.

**Synthesis of N,N‐diphenyl‐2‐vinylaniline (vinyltriphenylamine, VTPA)**. Methyl triphenylphosphonium bromide (8 g, 22.3 mmol) was dissolved in dry THF (50 mL), cooled to 0 °C and *t‐*BuOK (2.5 g, 22.3 mmol) added under stirring. 4‐(diphenylamino) benzaldehyde (5 g, 18.3 mmol) in dry THF (30 mL) was slowly added in a dropwise fashion. After 2 h at 0 °C the mixture was allowed to reach room temperature and stirred for another 12 h before being stopped with methanol. After drying over MgSO_4_ and removal of solvents under reduced pressure the crude product was purified by column chromatography (*silica*, DCM:cyclohexane 10 : 1, v:v). Yield: 88 % (4.4 g). ^1^H NMR (250 MHz, CD_2_Cl_2_): δ: 7.40–7.23 (m, 6H), 7.20–7.01 (m, 8H), 6.72 (dd, J=17.6 Hz, 10.9 Hz, 1H) 5.69 (dd, J=17.6 Hz, 0.9 Hz, 1H), 5.21 (dd, J=10.9 Hz, 0.9 Hz, 1H).

**4‐(Chloromethyl)styrene (CMS)**. The commercially obtained compound was filtrated over activated alumina to remove the added stabilizer prior to polymerization.

**P(VTPA‐*co*‐CMS)**. Free radical polymerization was performed with the three monomer feed ratios 25 : 75, 50 : 50 and 75 : 25. The reaction shall be described with the example of a ratio of 50 : 50. VTPA (2.04 g, 7.5 mmol) was prepared in a *Schlenk* flask and dissolved in dry toluene (15 mL). CMS (1.06 mL, 7.5 mmol) and AIBN (24.6 mg, 0.15 mmol), the latter freshly recrystallized from methanol, were added and the mixture degassed via freeze‐pump‐thaw. Afterwards the reaction was heated to 70 °C and kept stirring for 48 h. The polymer was precipitated from cold methanol and collected by centrifugation until an off‐white polymer was attained. Yields: 62 % (VTPA/CMS=25/75), 53 % (VTPA/CMS=50/50), 77 % (VTPA/CMS=75/25). ^1^H NMR (250 MHz, CDCl_3_): VTPA/CMS=75/25: δ: 7.02 (br), 6.95 (br), 6.57 (br), 4.38 (br), 1.97 (br), 1.51 (br), 1.18 (br), 1.04 (br). VTPA/CMS=50/50: δ: 7.12 (br), 6.95 (br), 6.82 (br) 6.54 (br), 4.44 (br), 1.85 (br), 1.47 (br), 1.17 (br), 1.03 (br). VTPA/CMS=25/75: δ: 7.18 (br), 6.98 (br), 6.78 (br) 6.50 (br), 4.47 (br), 1.69 (br), 1.41 (br), 1.13 (br), 0.92 (br). SEC (THF, PS standards): VTPA/CMS=75/25: $\bar{M_{n}}$ =6900 gmol^−1^, $\bar{M_{w}}$ =13900 gmol^−1^, *Ð*=2.01. VTPA/CMS=50/50: $\bar{M_{n}}$ =11500 gmol^−1^, $\bar{M_{w}}$ =24800 gmol^−1^, *Ð*=2.15. VTPA/CMS=25/75: $\bar{M_{n}}$ =6000 gmol^−1^, $\bar{M_{w}}$ =14500 gmol^−1^, *Ð*=2.41 and $\bar{M_{n}}$ =4800 gmol^−1^, $\bar{M_{w}}$ =9000 gmol^−1^, *Ð*=1.88.

**P(VTPA‐*co*‐AMS)**. The general procedure will be explained at the example of P(VTPA‐*co*‐CMS) 25 : 75. After dissolving the polymer (1.5 g) in dry dimethylformamide (20 mL), sodium azide (1.95 g, 5 eq to estimated CMS amount) was added to the solution and the mixture stirred under inert atmosphere for 24 h. After dilution with chloroform and water the organic phase was washed with 50 % brine (3×40 mL) and water (3×40 mL). After drying over MgSO_4_ the solvent was removed *in vacuo*. To remove remaining traces of DMF the polymers were dissolved in large amounts of CHCl_3_, the solution warmed to 50 °C and *n*‐heptane added to create a solvent ratio of chloroform:*n*‐heptane of 2 : 1. Subsequently the solvents were slowly removed under reduced pressure. Again, the polymers were dissolved in a small amount of chloroform and precipitated from methanol. ^1^H NMR (250 MHz, CDCl_3_): VTPA/CMS=75/25: δ: 7.03 (br), 6.92 (br), 6.53 (br), 4.09 (br), 1.95 (br), 1.47 (br), 1.13 (br), 1.01 (br). VTPA/CMS=50/50: δ: 7.12 (br), 6.93 (br), 6.78 (br) 6.50 (br), 4.12 (br), 1.82 (br), 1.44 (br), 1.14 (br), 1.01 (br). VTPA/CMS=25/75: δ: 7.18 (br), 6.96 (br), 6.75 (br) 6.50 (br), 4.17 (br), 1.71 (br), 1.44 (br), 1.12 (br), 0.90 (br). SEC (THF, PS standards): VTPA/CMS=75/25: $\bar{M_{n}}$ =5200 gmol^−1^, $\bar{M_{w}}$ =12200 gmol^−1^, *Ð*=2.34. VTPA/CMS=50/50: $\bar{M_{n}}$ =12 600 gmol^−1^, $\bar{M_{w}}$ =31800 gmol^−^1, *Ð*=2.52. VTPA/CMS=25/75: $\bar{M_{n}}$ =9500 gmol^−1^, $\bar{M_{w}}$ =26000 gmol^−1^, *Ð*=2.74 (after Soxhlet with cyclohexane) and $\bar{M_{n}}$ =6000 gmol^−1^, $\bar{M_{w}}$ =11800 gmol^−^1, *Ð*=1.92. IR (ATR): $\tilde{\nu }$ [cm^−1^]=2096 (−N_3_, 75/25), 2093 (−N_3_, 50/50), 2093 (‐N_3_, 25/75).

**P(VTPA‐*co*‐FcS)**. Films of P(VTPA‐*co*‐AMS) copolymers were prepared by spin‐coating from 5 g/L solutions in toluene. After characterization in cyclic voltammetry with electrochemical crosslinking, films were thoroughly washed with acetonitrile and dried *in vacuo* before being placed in glass vials containing elemental copper (1.9 mg, 0.03 mmol) and being transferred into a glovebox. A solution of ethynylferrocene (5.3 mg, 0.025 mmol) and catalyst tetrakis(acetonitrile)copper(I) hexafluorophosphate (1.0 mg, 0.003 mmol) in DMSO (2 mL) was added to each vial and the mixtures kept standing for 72 h. Afterwards, vials were removed from the glovebox and washed thoroughly with fresh DMSO and acetonitrile before being blow‐dried in an argon stream and stored *in vacuo* before characterization.

## Conflict of interest

The authors declare no conflict of interest.

## Supporting information

As a service to our authors and readers, this journal provides supporting information supplied by the authors. Such materials are peer reviewed and may be re‐organized for online delivery, but are not copy‐edited or typeset. Technical support issues arising from supporting information (other than missing files) should be addressed to the authors.

Supporting Information
