# Supplementary material for: Electrochemical Characterization of Redox Probes Confined in 3D Conducting Polymer Networks
Source: Chemistry. 2021 Nov 25;27(68):17255–63. doi: 10.1002/chem.202103257 (PMC9298994; doi:10.1002/chem.202103257)
Supplement: Supplementary file 1 — Supporting Information [file CHEM-27-17255-s001.pdf]

Chemistry–A European Journal

Supporting Information

Electrochemical Characterization of Redox Probes Confined in 3D Conducting Polymer Networks

Chemistry–A European Journal

Supporting Information

Electrochemical Characterization of Redox Probes Confined in 3D Conducting Polymer Networks

Supporting information

Self-assembled monolayer (SAM). The 11-azidoundecanethiol was synthesized according to an adapted procedure.[1] All commercial chemicals were used as purchased unless otherwise stated. The SAM of 11-azidoundecanethiol was assembled on a clean Au/Si substrate which was used immediately after the Snowjet step. The preparation was performed in a N_2-atmosphere glovebox, in the dark, and at room temperature. The 1 mM solution of 11-azidoundecanethiol was dissolved in dried ethanol (max. 0,01 % H_2O) purchased from Merck. The substrate was immersed in this solution for 24 hours. Afterwards, the sample was cleaned in ultrasound bath in three steps: 1) 10 minutes in the SAM solution, 2) 10 minutes in clean ethanol (absolute), and 3) 5 minutes in clean ethanol (absolute). Finally, the sample was dried in argon flow.

Ethynylferrocene (EFc) clicking with azido-SAM. All commercial chemicals were used as purchased unless otherwise stated. EFc (97 %) was purchased from Sigma Aldrich. The azido-SAM was subjected to the click reaction with EFc immediately after the preparation. A 10 mM click solution was prepared from EFc dissolved in methanol (distilled with 3 Å of molecular sieve) with a copper (I) catalyst system. The copper (I) catalyst system within the 10 mM solution consisted of 2.5 mM of tetrakis(acetonitrile)copper(I)hexafluorophosphate ($[(CH_3CN)_4Cu]PF_6$, 97 %) purchased from Sigma Aldrich and 15 mM of copper (powder, <425 µm, 99.5 % trace metal basis). The azido-SAM was submerged in this click solution and the reaction proceeded for 24 hours under N_2-atmosphere of the glovebox, in the dark, and at room temperature. Occasional stirring of the click reaction set-up was done manually. After 24 hours, the submerged azido-SAM sample was cleaned in ultrasound bath in three steps: 1) 10 minutes in 10 mM click solution, 2) 10 minutes in clean methanol (p.a), and 3) 5 minutes in clean methanol (p.a). Finally, the resulting ferrocene (Fc) substituted SAM, mediated by clicking of EFc to the azido tail groups, was dried in argon flow .

Polarization Modulation-Infrared Reflection Absorption Spectroscopy (PM-IRRAS). The instrument used is a Bruker© PMA50 equipped with a Hinds™ Instruments PEM-100 controller. The polarizer angle was 0 ° while the incidence angle of the detector was set to 80 °. The measurements were performed at room temperature and atmospheric pressure. A specific wavenumber keyed into the PEM-100 controller helped to enhance less intense signals in the IR spectrum with the frequency at 84.259 KHz and a retardation of 0.5 λ. Each measurement was taken with 1000 scans, a resolution of 4 cm^{-1} and a KBR source unless otherwise stated.

Further copolymer characterization

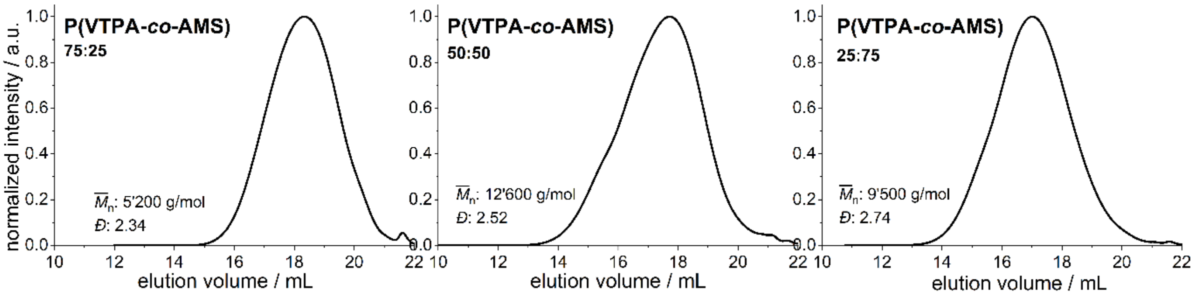

Figure S1. SECs of the azide-bearing copolymers measured in THF against polystyrene standards.

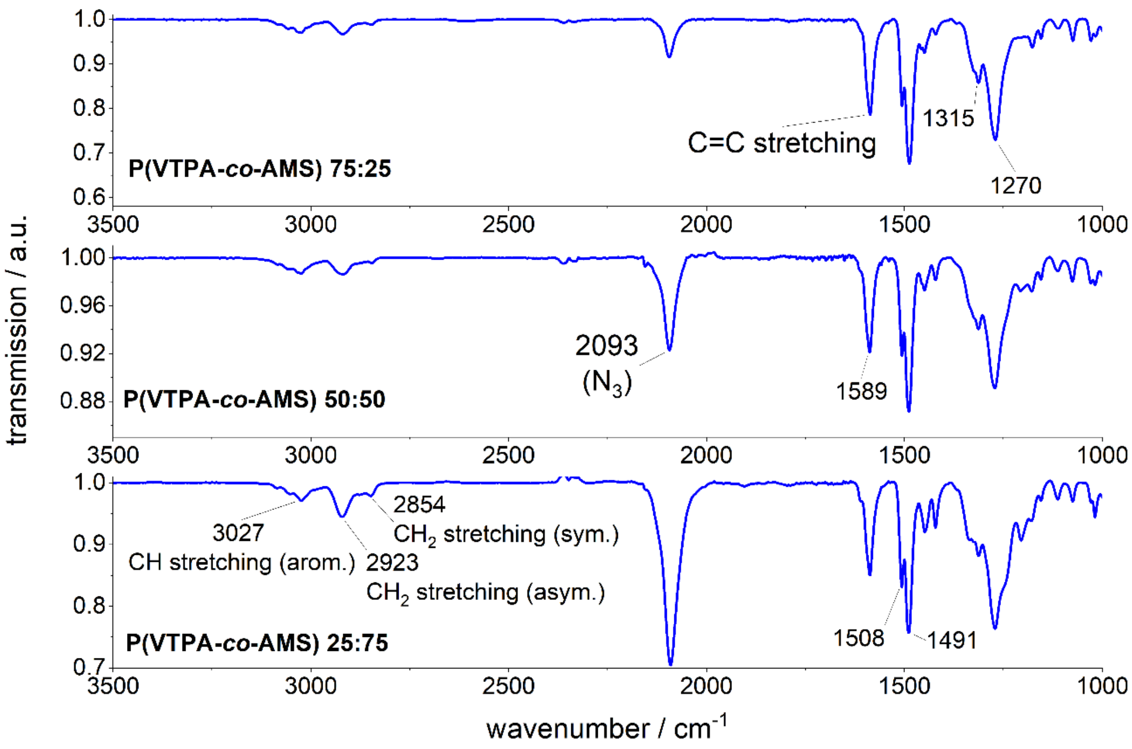

Figure S2. Attenuated total reflectance (ATR) infrared spectra of the azide-bearing bulk copolymers.

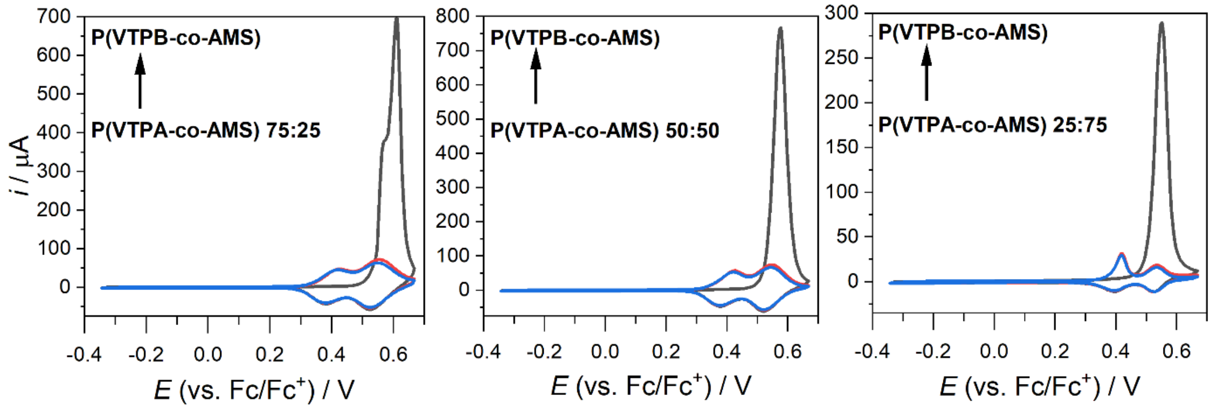

Figure S3. Electrochemical crosslinking of the azide-bearing copolymer films by cyclic voltammetry. Black: First cycles for the as-deposited films; coloured: Second and third cycles.

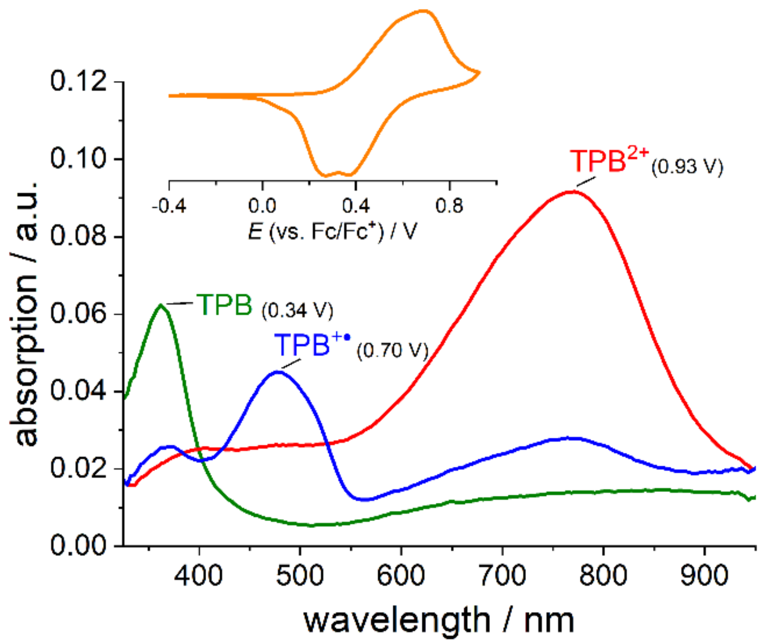

Figure S4. UV-vis spectra of a 25:75 polymer film on ITO taken at the specific voltages during the 3rd forward cycle after crosslinking and corresponding to the neutral, radical cation and dication TPB redox species. The respective CV cycle is given as the insert.

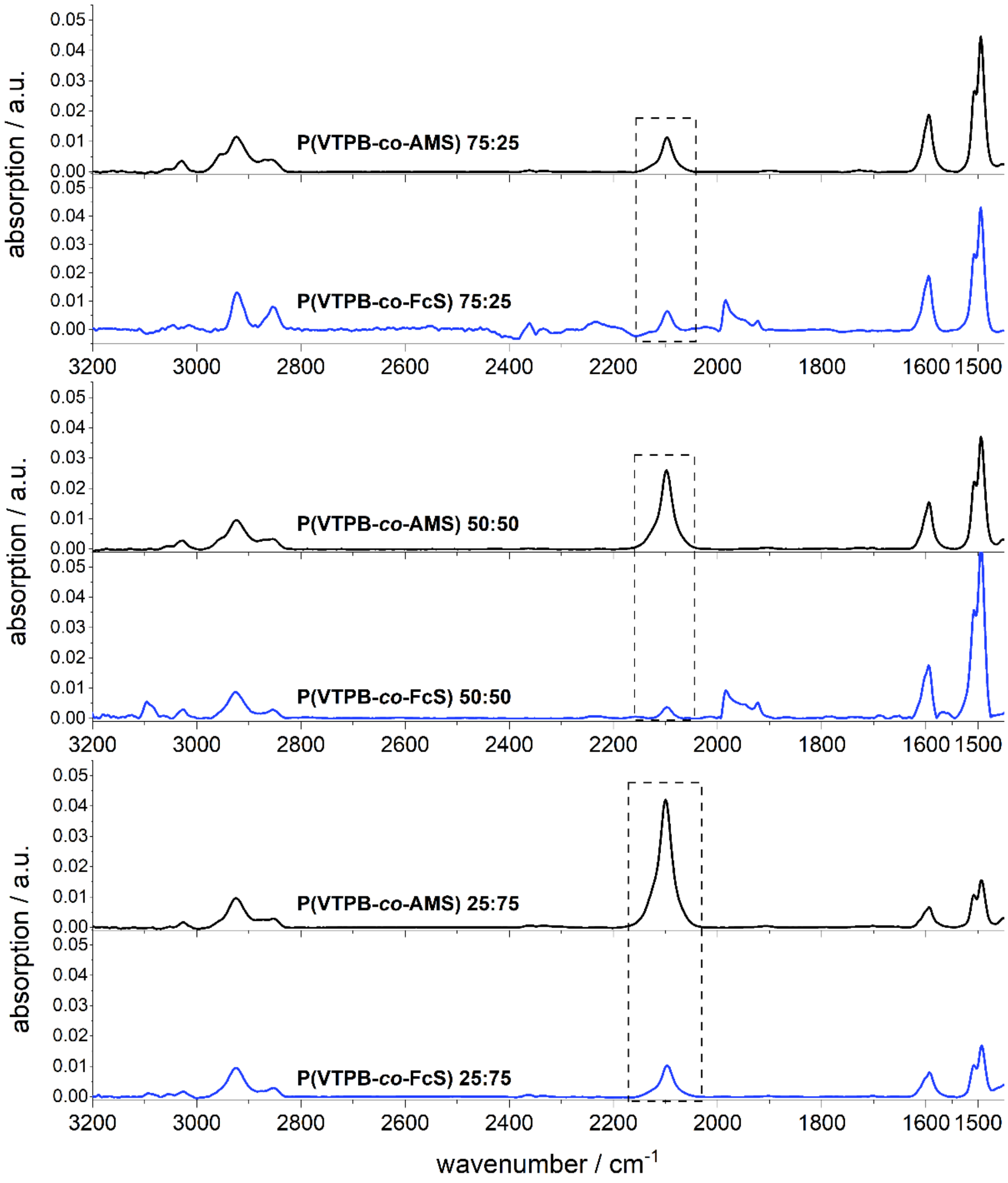

Figure S5. PM-IRRAS absorption spectra of the copolymer films before (black lines) and after (blue lines) CuAAC with ethynylferrocene. Characteristic azide bands are marked by black dashed boxes. The keyed wavenumber during the measurements was 2100 cm^{-1}.

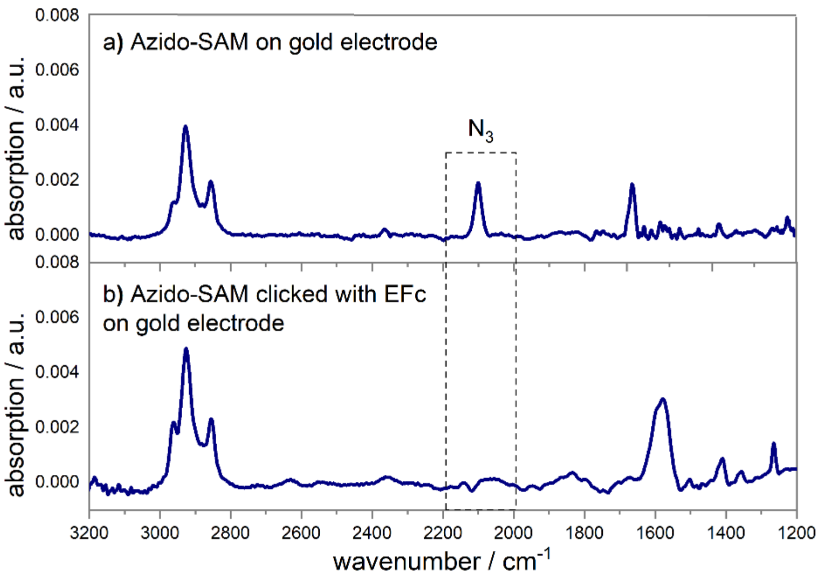

Figure S6. PM-IRRAS spectra of AUDT SAM on gold electrode before (a) and after (b) clicking with EFc. The successful clicking is emphasized by the disappearance of the characteristic azide (N_3) after the reaction.

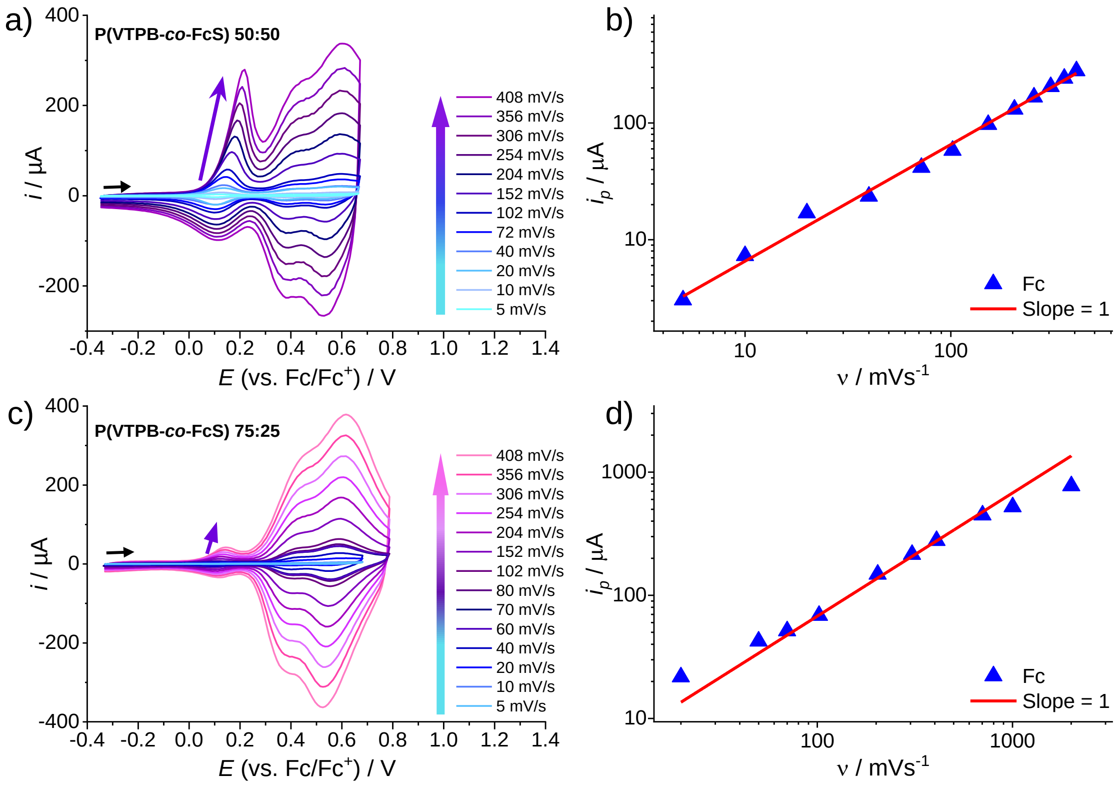

Figure S7. a) Cyclic voltammograms of 50:50 copolymer after CuAAC with EFc acquired at the different scan rates. b) Corresponding scan rate dependence of the forward scan peak current i_p of Fc. b) CV of 75:25 copolymer after CuAAC with EFc acquired at the different scan rates. b) Corresponding scan rate dependence of the forward scan peak current i_p of Fc. A linear relationship between i_p vs v are observed for both systems.

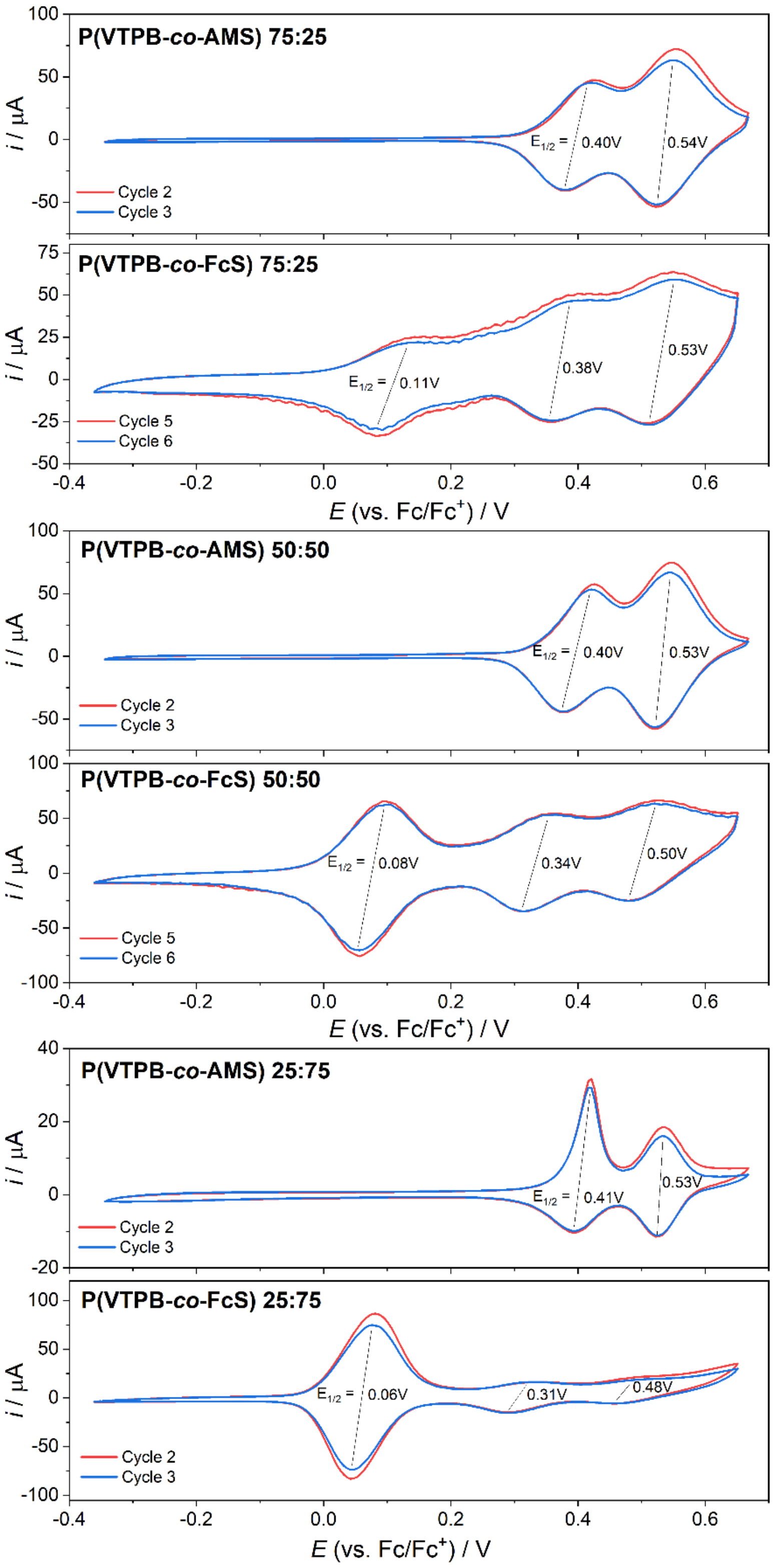

Figure S8. Cyclic voltammetry of the copolymer films performed after crosslinking (top panels) and after subsequent CuAAC with ethynylferrocene (bottom panels). The measurements were performed in $TBAPF_6$ in MeCN (0.1 M) at 50 mV/s.

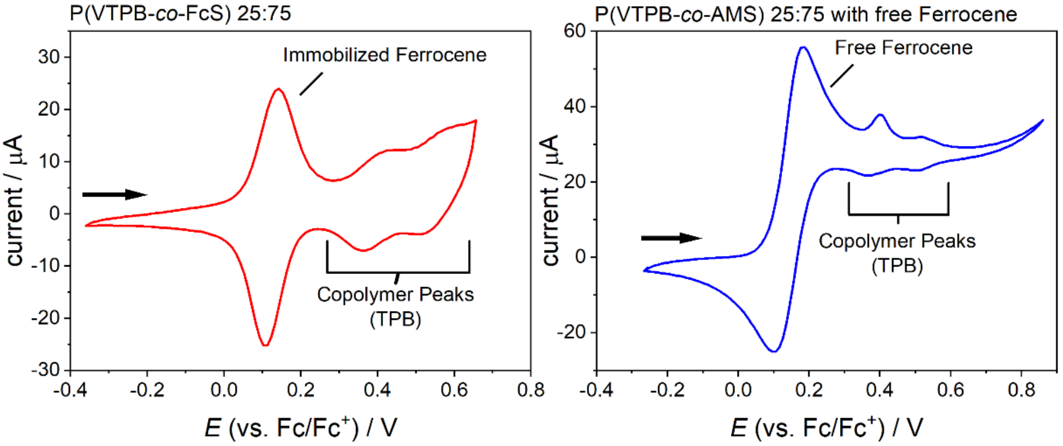

Figure S9. Comparative CVs of two copolymer films on gold. (Left) Polymer film with covalently bound ferrocene after click chemistry. (Right) Polymer film with loose ethynylferrocene added to the electrolyte solution with no click chemistry involved. $TBAPF_6$ in MeCN (0.1 M) at 50 mV/s (left) and 20 mV/s (right).

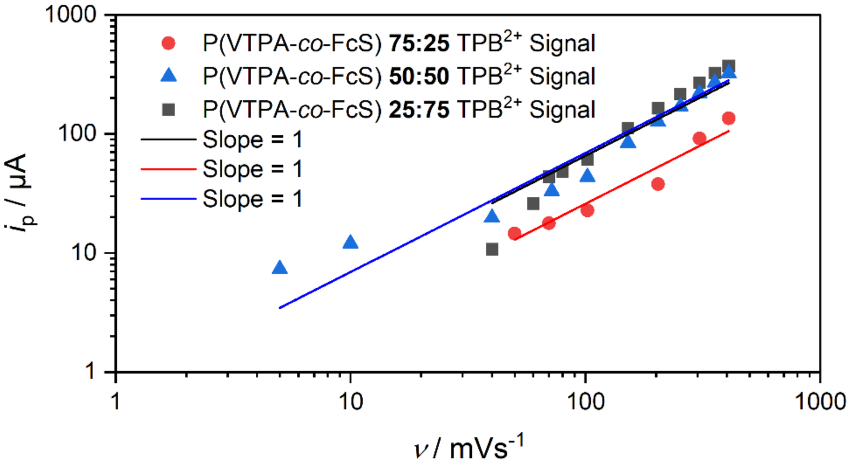

Figure S10. Scan rate dependence of the forward scans peak currents i_p for the $TPB^{\bullet+}$ → TPB^{2+} redox peaks. The linear relationship of i_p vs v is emphasized by linear fits of the experimental data.

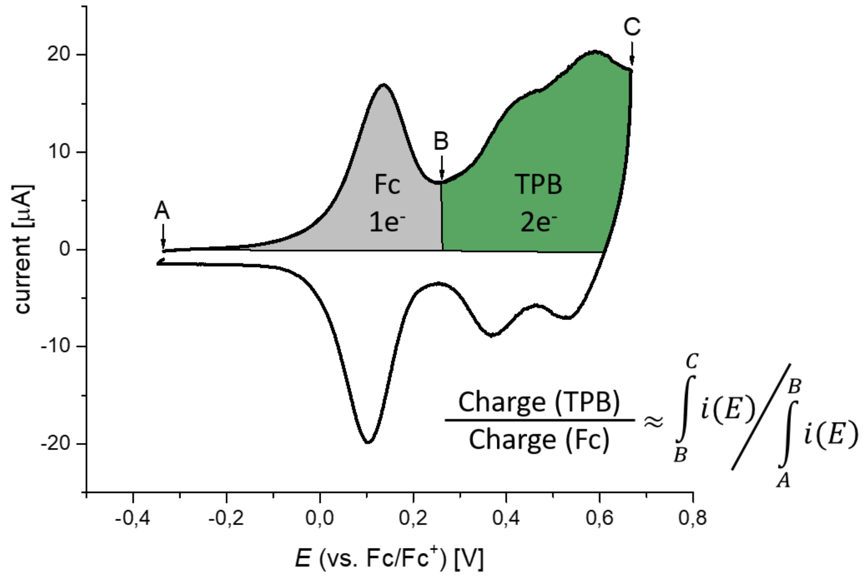

Molar Ratio Copolymer	25:75	50:50	75:25
Theoretical charge ratio (TPB/Fc)	0.67	2	6
Experimental charge ratio (TPB/Fc)	1.0	2.1	6.4

Figure S11. CV of the 25:75 copolymer film with highlighted integrals together with the calculations used to obtain charge ratios between TPB and Fc.

[1] A. Devadoss, C. E. D. Chidsey, *J. Am. Chem. Soc.* **2007**, *129*, 5370–5371.
